# On the Role of Iodine in Plants: A Commentary on Benefits of This Element

**DOI:** 10.3389/fpls.2022.836835

**Published:** 2022-03-22

**Authors:** Vitor L. Nascimento, Beatriz C. O. Q. Souza, Guilherme Lopes, Luiz R. G. Guilherme

**Affiliations:** ^1^Biology Department, Universidade Federal de Lavras, Lavras, Brazil; ^2^Soil Science Department, Universidade Federal de Lavras, Lavras, Brazil

**Keywords:** biofortification, essential element, metabolism, nutrition, transporters

Iodine (I) is one of the least abundant elements on Earth’s surface; soils have only about 3 mg kg^–1^ of total I (Mohiuddin et al., 2019). However, this value can be higher in places close to the coast and lower in areas with slight marine influence (Fuge and Johnson, 2015). The marine environment is rich in this element, having about 60 μg L^–1^ and being the largest I reservoir on the planet (Wong, 1991). Regarding availability of I in soils, a small amount of it is present in the soil solution, with the major fraction being associated with the solid phase, i.e., organic matter and clay minerals, as well as iron (Fe) and aluminum (Al) oxides (Fuge and Johnson, 1986). Some substrate characteristics, such as mineral/organic composition, pH, texture, and redox conditions, limit I mobility and, thus, its absorption by plants (Gonzali et al., 2017). Consequently, knowing the distribution of I worldwide is key for a better understanding of its importance in living beings, from microorganisms to humans, and in plants.

Iodine is an essential element for animals, being involved in regulation of growth, development, and metabolism (Blasco et al., 2008), as it is required for the synthesis of thyroid hormones (thyroxine and triiodothyronine) (Landini et al., 2012). According to Dai et al. (2004), I deficiency in humans can cause a series of diseases and health problems, such as goiter, cretinism, reduced intellectual capacity, spontaneous abortions in pregnant women, congenital defects in fetuses, and deaths in babies at birth. Iodine’s bioavailability in food is considered high (∼99%) (Weng et al., 2013). However, some factors, such as food preparation and storage, among others, can affect the bioavailability of I in the human body, causing its deficiency (Gonzali et al., 2017). In the marine environment, algae (especially brown algae) and phytoplankton are I hyperaccumulators, helping to convert iodate (IO_3_^–^) into iodide (I^–^), the most absorbable form for terrestrial plants (Chance et al., 2007). The importance of I in plants has not yet been fully explained, but application of I^–^ in plant species has provided greater accumulation of the element in edible parts of lettuce (*Lactuca sativa*), spinach (*Spinacia oleracea*), and curly endive (*Cichorium endivia* L. var. *crispum* Hegi) (Zhu et al., 2003; Weng et al., 2008a; Blasco et al., 2013; Smoleń et al., 2016; Sabatino et al., 2021), as well as rice (*Oryza sativa*), wheat (*Triticum aestivum*), and maize (*Zea mays*) (Cakmak et al., 2017), because this process of biofortification is an affordable way to avoid I deficiency in human populations (Blasco et al., 2008, 2013; Prom-u-thai et al., 2020), especially when I is applied as potassium iodate (KIO_3_) (Cakmak et al., 2017). Plants, algae, and phytoplankton are also capable of volatilizing I in the form of iodomethane (also known as methyl iodide, CH_3_I), and this reaction is catalyzed by enzymes with methyltransferase activity dependent on S-adenosyl-L-methionine (Itoh et al., 2009). Volatilization is possibly associated with defense function while also serving to aid in global I cycle (Fuge and Johnson, 2015; Gonzali et al., 2017). However, emissions, both terrestrial and marine, can contribute to the damage in the ozone layer, with impacts on the stratosphere still uncertain (Koening et al., 2020). Thus, it is seen that I plays a substantial role in metabolism in animals and especially in humans, while its importance in plants has not yet been fully unveiled. However, what is known so far about the relationship of I with plants?

Iodine can be absorbed by plants from the soil solution *via* roots and through the air, by rain, or dissolved in saline solutions (Kiferle et al., 2021), entering across the stomata and cuticular layer of leaves (Whitehead, 1984). Absorption of I is more efficient through hydroponic systems than *via* soil applications (Smoleń et al., 2016), and both are apparently more efficient ways to supply I to plants than foliar sprays. However, the use of surfactants can increase the absorption of element through this technique (Lawson et al., 2015; Gonzali et al., 2017). Anyhow, more studies are needed to define the best methods for delivering I more efficiently to different crops. Through soil, I is transported into plants by H^+^/anion symporters in cells of roots, following the same pathway as chloride (Cl^–^) (White and Broadley, 2009). However, the molecular identity of these specific transporters has not yet been unveiled. Despite this, it is suspected that homologs of band 3 anion transporter (also known as anion exchanger 1—AE1) also carry I^–^ (Bruce et al., 2004), as well as specific Cl^–^ channels that are immediately permeable to these anions (Roberts, 2006). These channels are encoded by chloride channel (CLCs) transporter genes, which have family members that are I-permeable H^+^/anion antiporters (White and Broadley, 2001; Nakamura et al., 2006). These same antiporters, together with anion channels in the tonoplast (i.e., lipoprotein membrane that limits the vacuoles), are likely to transport I^–^ into and out of the vacuoles in plant cells (De Angeli et al., 2006). Furthermore, halide (i.e., chemical compounds of the same family as I) fluxes can be facilitated by organic acid transporters (White and Broadley, 2001). Thus, it is only a matter of time for specific I^–^ transporters to be identified and their forms of action to be described. Moreover, in plant tissues, I accumulate in the vacuoles, and in a systemic view of plants, the accumulation process goes from roots to leaves, and then to stems (Weng et al., 2008a). Inside plant tissues, the inorganic form of I, mainly I^–^, is predominant (Weng et al., 2008c), but it can also be absorbed as IO_3_^–^ (Fuge and Johnson, 1986). However, plants can absorb organic molecules in the form of iodosalicylates, iodobenzoates, monoiodotyrosine, diiodotyrosine, and triiodothyronine (Smoleń et al., 2020). This element has a predominantly xylem movement in plants, but a phloem route has been identified in tomato (*Solanum lycopersicum*) and lettuce (Landini et al., 2012; Smoleń et al., 2014). Therefore, knowing the role of I in plants at the level of absorption and internal movement is key for establishing effects that this element has on plant nutrition, metabolism, and, consequently, on growth.

Iodine shows evidence of having a role in the metabolic process of plants, as demonstrated by Kiferle et al. (2021), where it increased biomass production and anticipated the flowering of Arabidopsis (*Arabdopsis thaliana*); it was also present in root and shoot proteins, and helped to modulate the expression of genes involved in defense responses. Some authors, such as Lehr et al. (1958) and Borst Pauwels (1961), six decades ago, considered I as a micronutrient for plants because when applied in small amounts it is related to positive effects on some plant species, such as those previously mentioned, in addition to the fact that I increase production of components of the antioxidant system in lettuce plants, as reported by Blasco et al. (2008). Nevertheless, when applied in high amounts, I can cause symptoms of toxicity, such as leaf lesions, stunted plant growth, and, ultimately, plant death (Lehr et al., 1958; Weng et al., 2008b). These factors make I comparable to other plant micronutrients, such as boron (B), chlorine (Cl), copper (Cu), Fe, manganese (Mn), molybdenum (Mo), nickel (Ni), and zinc (Zn). However, to what extent is this comparison applicable? Although several studies are being carried out to unravel the effects of I on plants, there is still lack of evidence that I can be considered a (micro)nutrient for plants instead of being just a beneficial element.

Brown et al. (2021), in a review addressing the concept of plant nutrients and their evolving definitions, provided a historical perspective on the conceptualization of essential elements for plants. One of the most important concepts was established by Arnon and Stout (1939), who considered that an element would only be essential if: “(*i*) its deficiency makes it impossible for the plant to complete the vegetative or reproductive phase of its life cycle; (*ii*) its deficiency is specific to the element in question, and can be prevented or corrected with its supplementation; and (*iii*) the element is directly involved in plant nutrition, regardless of its possible effects in the correction of any unfavorable microbiological or chemical condition of the soil or other culture medium.” Over time, these definitions have changed slightly, and one of the most recent concepts is in the first chapter of the book “Mineral Nutrition of Higher Plants” (Kirkby, 2012), where for an element to be considered essential it must meet the following three requirements “(*i*) a plant should be unable to complete its life cycle in the absence of the element; (*ii*) the element’s function must not be replaced by another element; and (*iii*) the element must be directly involved in plant metabolism, as a component of an essential plant constituent, such as an enzyme, or it must be required by a distinct metabolic step, such as an enzymatic reaction.” According to Broadley et al. (2012), differentiation between beneficial and essential elements is difficult, especially in the case of trace elements, such as I. Whitehead (1984) considers I as a non-essential element, while more recent studies by Sahin (2020) and Medrano Macías et al. (2021) address I as a non-essential but beneficial element. With passage of time and occurrence of a greater number of studies on this subject, it is natural that more elements are added to the list of essentials (or beneficial) elements established by the previously mentioned authors. The fact that I is currently included in the list of beneficial elements shows an evolution of pre-established definitions and standards. These concepts are increasingly being adjusted to the realities of modern agriculture and to the needs of tackling micronutrient deficiencies in human populations (hidden hunger).

Thus, in this scenario of constant change, an ongoing debate on features and definitions of elements’ essentiality becomes necessary, as science is done every day, and new research data are always being published. For now, attentiveness is needed, as there is still a lot to be known about the effects, in the short and long terms, of I on plants, and its exogenous application not only in plants but also in the environment. In conclusion, it is fascinating that in 2022 we are still discovering the functionality of a chemical element in plant nutrition and metabolism. Here, we do not discard the possible essentiality of this element; however, we highlight that the findings in Arabidopsis need to be demonstrated in a greater number of plant (crop) species. Iodine is perhaps not an essential element, but it is beneficial as silicon (Si), selenium (Se), sodium (Na), and others. Finally, our position is in support of efforts to promote crop biofortification with this element to tackle hidden hunger.

## Author Contributions

All authors conceived the idea, collected the data, and contributed to writing the article.

## Conflict of Interest

The authors declare that the research was conducted in the absence of any commercial or financial relationships that could be construed as a potential conflict of interest.

## Publisher’s Note

All claims expressed in this article are solely those of the authors and do not necessarily represent those of their affiliated organizations, or those of the publisher, the editors and the reviewers. Any product that may be evaluated in this article, or claim that may be made by its manufacturer, is not guaranteed or endorsed by the publisher.
